# School-based indicated prevention interventions for anxiety in children and adolescents: A commentary on a systematic review

**DOI:** 10.12968/chhe.2024.5.3.122

**Published:** 2024-07-26

**Authors:** C Harris, D Packwood, A Wood, L Loughran, P Wheeler, J Hill

**Affiliations:** University of Central Lancashire; Voluntary Sector North West; The University of Liverpool; Koala North West; Lancaster University; University of Central Lancashire

## Abstract

Mental health problems such as anxiety are on the increase in children and adolescents. However, rising demand and cuts to mental health services make it difficult for young people to access the support they need. School-based interventions aimed at preventing or reducing anxiety have the potential to provide help to large numbers of children and adolescents. However, the effectiveness of these interventions for different groups is somewhat unclear. This commentary summarises and critically appraises a recent systematic review which investigated the effectiveness of school-based interventions to prevent and reduce anxiety symptoms in indicated groups of children and adolescents.

## Introduction

Whilst the NHS Long Term Plan (NHS 2019) has increased investment into Children and Young People’s mental health services, rising prevalence and demand continue to impact on access and waiting times for mental health services (NHS Digital 2022). During the COVID-19 pandemic, there was a deterioration of mental health and an increase in depression, anxiety and psychological distress in children and adolescents (Kauhanen et al. 2023). Additionally, since the pandemic there has been an increase in children and adolescents being absent from school with reports suggesting that anxiety is a factor (The Centre for Social Justice 2023). There is therefore likely to be an increased need for interventions to reduce anxiety symptoms and prevent anxiety disorders in these populations in the coming years.

However, waiting lists and cuts to mental health services make it increasingly difficult for young people to access the support they need (Young Minds 2023). McGorry, Mei, and Chanen et al. (2022) argue that despite adolescents having the greatest need, they have the worst access to timely and quality mental health care. Schools are well placed as a setting to deliver early and preventative mental health interventions to large numbers of children and adolescents thereby increasing access to support (Masia-Warner et al. 2006). However, the effectiveness of school-based interventions aimed at preventing or reducing anxiety for different groups (universal, targeted or indicated) is somewhat unclear (Neil and Christensen 2009). A recent systematic review by Hugh-Jones et al., 2021 aimed to update and synthesise evidence on the effectiveness of school-based interventions to prevent and reduce anxiety symptoms in indicated groups of children and adolescents.

### Aim of commentary

This commentary aims to critically appraise the methods used within the review Hugh-Jones et al., 2021 and expand upon the findings in the context of clinical practice.

## Methods

The authors carried out a robust search of six databases from date of inception to December 2019, which was supplemented by reference checking of included studies and a search for grey literature using Google Scholar. The review included randomised controlled trials (RCTs) of school-based interventions for indicated prevention/early-intervention of anxiety disorders in children and adolescents (aged 5-18) who had elevated symptoms of anxiety. Comprehensive screening, data extraction, and risk of bias assessment processes were undertaken independently by at least two reviewers. Risk of bias assessment was conducted using the Cochrane Collaboration’s tool for assessing risk of bias. The main outcome assessed was reduction of anxiety symptoms measured by either self-rated or clinician-rated scales, or diagnostic interviews. A meta-analysis was conducted using a random-effects model. Heterogeneity was assessed through use of the *I*^*2*^ statistic. Publication bias and small study effects were assessed through use of a funnel plot, with Egger’s tests conducted where asymmetry was observed. Sub-group analyses were conducted for comparisons between types of intervention (cognitive behaviour therapy or non-cognitive behaviour therapy) and control group (waitlist, no intervention, or attention control), and for intervention intensity (delivered weekly, biweekly or twice weekly).

## Results

The searches identified 2547 studies after duplicates were removed. Following the screening processes 20 studies were included in the review, of which 18 were included in the meta-analysis. The risk of bias was assessed as being high across the included studies. This was due to inadequate reporting of randomisation procedures in most of the included studies, risk of contamination between groups randomised within the same schools in the majority of the studies, and difficulties with blinding participants and personnel in 75% of the studies. It was also not possible to blind the outcome assessment in 70% of the studies where the outcomes were self, or parent assessed. There was an unclear risk of reporting bias in nearly all the studies.

The review found that overall, school-based indicated prevention programmes produced a statistically significant reduction in anxiety symptoms in participants (standardised mean difference of −0.28, 95% Confidence Interval −0.50 to −0.05) based on the results of 18 studies. Substantial heterogeneity was found for the overall effect estimate (I^2^=78%). The review reported the effect of the interventions at different follow-up periods and found that there was a reduction in anxiety symptoms in the first six months after the interventions based on the results of nine of the studies (SMD=−0.35, 95% Confidence Interval of −0.58 to −0.13). Based on four of the studies a beneficial effect was still observed at 6-12 months (SMD=−0.24, 95% Confidence Interval of −0.48 to 0.00). However, the effect was not maintained in the long-term with no statistical difference between the intervention and control groups found after 12 months, based on the results two of the studies (SMD=−0.01, 95% Confidence Interval of −0.38 to 0.36).

Furthermore, sub-analyses comparing different intervention and control types only showed a significant effect when comparing cognitive behaviour therapy (CBT) interventions to a wait list control (SMD=-38, 95% Confidence Interval -0.74 to -0.02). Other comparisons (CBT vs attention control, CBT vs no intervention, non-CBT vs wait list, non-CBT vs attention control) did not show statistical difference between the intervention and control groups. The sub-analyses also showed that there were no significant differences on the size of the effect between the different types of control group used as a comparator, or between the different levels of intervention intensity (if interventions were delivered weekly, biweekly or twice weekly).

### Commentary

The AMSTAR2 tool was used to assess the quality of the review, and 12 out of 16 criteria were found to be satisfactory. Overall, we believe the review to be of moderate quality and it may provide an accurate summary of the results of the available studies which address the question of interest. However, the review would have benefitted from further information in the following domains. Although the authors performed a comprehensive search of multiple databases, supplemented by complementary search techniques, they did not consult experts in the field which could have helped to identify further studies which may have been missed in the database searches (McManus et al. 1998). The authors also did not provide a list of the studies that were excluded, although they did provide reasons for the exclusions. Without knowing which studies were excluded it is difficult to assess the impact of their exclusion from the review (Shea et al. 2017). The review authors also did not report on the sources of funding for the studies included in the review, which is important to consider as the design, conduct, analysis, and reporting of a trial can potentially be influenced by conflicts of interest with funders (Boutron et al. 2023). Furthermore, the findings of the review are limited by a lack of detail around participant characteristics. Although the authors described the ages of the participants, they did not report any information relating to participants’ ethnicity, gender or socioeconomic factors, and there was no detail relating to other child and adolescent vulnerabilities such as learning difficulties or additional needs. This makes it difficult to generalise the findings across diverse populations of children and adolescents in different school environments.

Based on the evidence in this review, school-based indicated prevention interventions may have a small positive effect on reducing symptoms of anxiety in children and adolescents. However, there was substantial heterogeneity in the effect estimate, as well as concerns regarding the risk of bias in the included studies, which reduces certainty in the estimate of effect. Schools in England are expected to recognise emerging wellbeing issues in students and to help students access early support and interventions (Public Health England 2021). School leaders and commissioners of mental health services could therefore consider offering group interventions for the prevention of anxiety disorders in indicated children and adolescents with elevated symptoms of anxiety.

However, given the potentially small size of effect estimated in the review, consideration should be given to the likely level of impact and cost-effectiveness of these interventions if they were to be delivered at scale. A recent review of economic evaluations found weak evidence that selective and indicated cognitive behaviour therapy interventions might be cost effective for preventing anxiety disorders in children and adolescents but concluded that as only a small number of economic evaluations have been conducted further research is needed to strengthen the evidence base (Anna-Kaisa et al. 2022).

Most of the studies (n=16) in the review explored cognitive behaviour therapy (CBT) based interventions. Only four studies looked at non-CBT based interventions and there is uncertainty around the effectiveness of these types of interventions. Non-CBT interventions may be less costly to deliver than CBT-based interventions (Richards et al. 2017), and so further research is needed to better establish their clinical and cost effectiveness.

The review focussed on interventions delivered in school-based settings. However, interventions delivered in other community settings may also be beneficial, particularly where children have anxiety around attending school. The Voluntary, Community, Faith and Social Enterprise (VCFSE) sector has been working in the mental health field for years. For example, UK based charities such as Childline (Childline n.d.) which offers advice, support and guidance for children and young people over the telephone, or Place2Be (Place2Be n.d.) which offers therapeutic interventions in primary school settings. There is currently very little research on mental health interventions delivered by the VCFSE sector or delivered in other settings in the community. A recent scoping review of barriers and facilitators to implementation of interventions for mental health prevention, promotion, and treatment in children in the UK did not identify any studies conducted in a community setting (Thomson et al. 2023). Further research investigating interventions delivered by the VCFSE sector, and in non-school settings, is therefore needed to assess the effectiveness of these services.

The studies included in the review highlight a variation in outcome measurement tools used which makes it difficult to accurately estimate effects across studies. Standardisation of outcome measures in future research would be beneficial so that the effectiveness of these interventions can be more precisely measured and compared. Work on this is currently being developed as the Core Outcomes and Measures in Pediatric Anxiety Clinical Trials (COMPACT) Initiative has pre-registered a study to develop an evidence- and consensus-based core outcome set for paediatric anxiety disorders for use in future clinical trials (Monga et al. 2023).

Finally, more research is needed to assess the effectiveness of interventions in the longer term. Few of the studies included in the review assessed the effects of the interventions at follow-up periods beyond the first six months, and only two studies assessed the effects after 12 months. Therefore, future studies should incorporate longer follow up periods to assess the efficacy of the interventions over longer periods of time.

### CPD reflective questions

What are the strengths and weaknesses of this systematic review?

Based on the evidence this review, do you think that school-based interventions should be offered to children and adolescents with elevated symptoms of anxiety?

What factors do you think should be considered when implementing school-based mental health interventions?

## Key Points

- Based on the evidence in the review school-based interventions may have a small positive effect on reducing symptoms of anxiety in indicated groups of children and adolescents.
- Given the potentially small estimate of effect size, further research is needed to evaluate the cost-effectiveness of school-based prevention interventions.
- The effectiveness of interventions delivered in other community settings should be assessed in future research.
